# Holding it together: Naa60 at the Golgi

**DOI:** 10.18632/oncotarget.4779

**Published:** 2015-07-02

**Authors:** Henriette Aksnes, Michaël Marie, Thomas Arnesen

**Affiliations:** Department of Molecular Biology, University of Bergenand and Department of Surgery, Haukeland University Hospital, Bergen, Norway

Since its first description over a century ago, the Golgi apparatus has received a great deal of interest. Despite considerable advances in identifying key components during the past decades, the molecular mechanisms underlying the maintenance of the structural organization and functional integrity of this dynamic organelle are not yet really understood. Our laboratory recently proposed that Naa60 might be a player in these processes since we found Naa60-depleted cells to suffer structural abnormalities of the Golgi apparatus [1]. Naa60 (Nα-acetyltransferase 60) belongs to a family of enzymes, the N-terminal acetyltransferases (NATs) that catalyze acetylation of the Nα-group that is present on all protein N-termini (Nt-acetylation) [2]. For several proteins, an acetylated N-terminus is essential either for its half-life, subcellular targeting or interaction properties [3, 4]. At the cellular level, Nt-acetylation has emerged as a crucial protein modification since NAT-lacking cells suffer severe malfunctions [4]. Also, the physiological significance of Nt-acetylation has become apparent through recently discovered diseases caused by genetic defects or deficiencies in NAT enzymes [4]. But still we know little about how the NATs perform these tasks at the molecular level.

Our recent study [1] demonstrated Naa60 to be an original among the NATs. Firstly, Naa60 (also called NatF) drastically differed from the other known human NATs in terms of its subcellular localization. Naa60 had an organellar localization, mainly to the Golgi, whereas the other NATs had cytosolic and cytosolic/nuclear localization patterns. Secondly, we found Naa60 to preferentially Nt-acetylate transmembrane proteins. This is a completely new concept in NAT substrate specificity, which is generally defined by the few first N-terminal amino acids, not by the protein's residency. Of notice, we found Naa60 to act on protein N-termini facing the cytosolic side of various intracellular membranes and the topological position of Naa60 matched accordingly [1]. Thirdly, the abovementioned features of Naa60 indirectly suggest that it might diverge from the established dogma of co-translational enzymatic activity for the NATs. Based on these characterizations of Naa60 we entitled it the first organellar N-terminal acetyltransferase.

Whereas more than 80% of all soluble human proteins have previously been estimated to be Nt-acetylated [5], transmembrane proteins have been a thus far poorly characterized part of the Nt-acetylome. Interestingly, in addition to identifying particular transmembrane proteins as substrates of Naa60, we further showed that Nt-acetylation turns out to be a frequent modification also among transmembrane proteins [1]. Thus this work emphasizes the widespread nature of this somewhat under-acknowledged protein modification by demonstrating its applicability also to the transmembrane and organellar proteins.

In addition to Naa60's presence at the Golgi, our study [1] also indicated an important role of Naa60 related to the structural integrity of this organelle. Knockdown of Naa60 caused the Golgi to fracture into smaller, more dispersed substructures, suggesting these cells to lack the structural organization that is referred to as the Golgi ribbon. This higher-ordered structural entity is made up of multiple cisternal stacks that are connected laterally into a ribbon-like structure near the perinuclear region. Additional confocal fluorescence microscopy analysis of Naa60-deficient cells revealed that *cis* and *medial* Golgi marker proteins remained in close proximity, hence suggesting cisternal stacking to be preserved, but the ribbon to be unlinked and dispersed into mini-stacks.

The morphological organization of the Golgi apparatus varies among different cell types and species, with the ribbon-shaped structure typically appearing in vertebrates, whereas lower eukaryotes typically have a less complex organization of their Golgi [6]. Interestingly, Naa60 also has a similar evolutionary profile, as it is absent from yeast and unicellular eukaryotes. Conceivably, Naa60 could have co-evolved with the increased complexity of the Golgi. The functional advantage of the linked ribbon structure is still uncertain. It is not required for general membrane trafficking and secretion, and a potential role in glycosylation is unclear, however it is essential in polarization of the secretory pathway [6].

**Figure 1:**
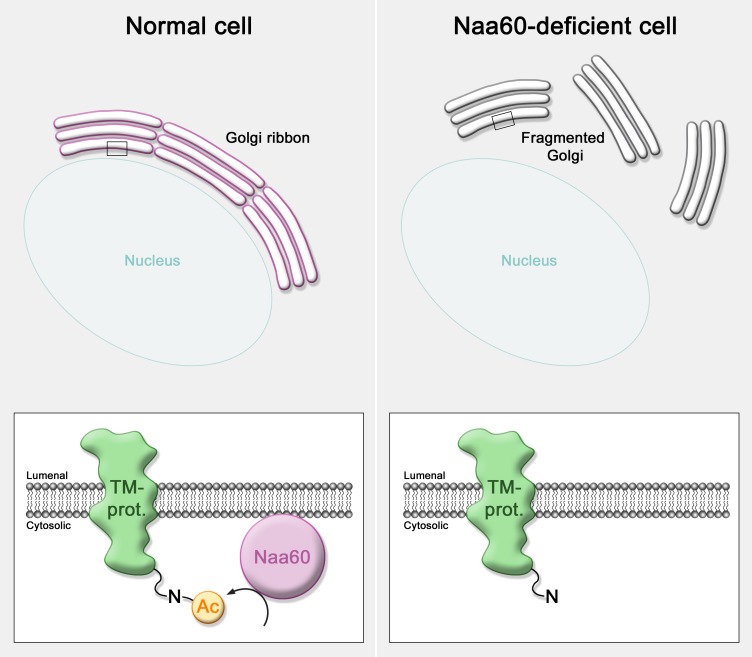
A model of Naa60's function in Golgi ribbon maintenance This model is based on the observation that Naa60-knockdown causes fragmentation of the Golgi ribbon [1] and proposes that Naa60 may function in maintaining the Golgi ribbon through acetylation of N-termini of transmembrane proteins with crucial functions to the Golgi.

In terms of understanding the function of Naa60 at the Golgi, it is interesting to consider which proteins might potentially require the Nt-acetyl group that is provided by Naa60. A large number of tethering factors and linker proteins contribute directly to the stacking of Golgi cisternae and formation of the Golgi ribbon [7]. Also, the dynamic nature of the Golgi makes the balance of membrane trafficking, and thus proteins of the transport machinery, essential in the maintenance of Golgi structural organization [7]. In addition, Golgi positioning and organization depend on microtubules and motor proteins [7]. How the presence of Naa60 is essential for the property of the Golgi to form or maintain the ribbon structure is yet unanswered. However, based on available data, it seems reasonable to infer that it may involve the Naa60-mediated Nt-acetylation of membrane proteins with key functions in this process. The proposed model of Naa60 functioning is shown in Figure 1.
